# Functional and nonfunctional parathyroid carcinoma: two case reports and literature review

**DOI:** 10.1007/s12672-023-00841-w

**Published:** 2023-11-29

**Authors:** Zhidong Yin, Xi Xu, Lu Cheng, Weike Kong, Yingfei He, Xiaogang Wang

**Affiliations:** Department of Pathology, Second Affiliated Hospital, Zhejiang University School of Medicine, No. 88 Jiefang Road, Hangzhou, 310009 Zhejiang China

**Keywords:** Parathyroid carcinoma, Pathological characteristics, Diagnosis, Operation

## Abstract

Parathyroid carcinoma (PC) is a rare malignant endocrine tumor. It can be divided into functional and non-functional types according to the tumor’s ability to secrete parathyroid hormone. Herein, we present a case each of functional and nonfunctional PC. Case 1: Functional PC. The main clinical symptoms were high parathyroid hormone and hypercalcemia with bone injury and other complications. The mass was large, capsulated, and showed vascular invasion. The tumor was surgically removed, along with a part of the tracheal wall and recurrent laryngeal nerve that were invaded by the tumor. The ipsilateral and isthmus thyroid lobe and central lymph nodes were also removed. Medicines were given to lower blood calcium. The patient died 18 months after surgery because of severe pulmonary infection and tracheal stenosis. Case 2: Non-functional PC. The patient showed no obvious clinical symptoms, but physical examination revealed a thyroid nodule. Despite the small diameter, the mass still invaded the surrounding thyroid lobe, fat, and muscle tissue. Surgery was performed to remove the tumor and ipsilateral thyroid lobe and central lymph nodes. The patient survived without recurrence or metastasis. Thus, we believe that the prognosis of PC negatively correlates with the scope of surgery. Early surgery can improve patient prognosis, and physical examination is conducive to early detection of PC. Herein, we provide a description of the diagnostic workup and the treatment approach and review relevant studies. We summarize the clinicopathological characteristics of PC cases to provide evidence for early diagnosis and therapy, to improve patient prognosis.

## Introduction

Parathyroid carcinoma (PC) is a rare endocrine malignancy with a very low incidence rate, accounting for 0.005% of all malignancies and < 1% of all primary hyperparathyroidism (PHPT) cases [1, 2]. However, the incidence rate has shown an upward trend [3]. The age of onset is usually 40–50 years, and the incidence rate is not related to sex [1, 2]. The etiology of PC is presently not clear. The clinical symptoms of patients with PC are mainly related to whether the tumor secretes parathyroid hormone (PTH). Approximately 90% of PCs have secretory function and present with signs and symptoms of hypercalcemia. Imaging helps not only to localize the disease but also to detect recurrence and metastasis. Ultrasonography and Technetium-99 m methoxy-isobutyl-isonitrile (Tc-99 m MIBI) or a 4-dimensional computed tomography (CT) scan are the imaging modalities of choice to characterize the lesion as well localize the tumor. In imaging examinations, PC mostly show up as large-volume tumors (tumor diameter, > 3 cm) that are lobulated with uneven texture echo, irregular shape, unclear boundary, calcification in nodules, cystic degeneration, and local infiltration [1]. The definitive diagnosis of PC is usually dependent on histopathology. The World Health Organization criteria suggest the diagnosis of PC should be restricted to tumors that show evidence of invasive growth involving adjacent structures (thyroid or soft tissue), capsular and extracapsular blood vessels, perineural space, and/or to those with documented metastases [3]. Surgical resection is the preferred treatment for PC. In addition, medical adjuvant therapy such as normal saline, loop diuretics, calcitonin, and diphosphates is usually used for the treatment of hypocalcemia and related complications. The first operation of PC should completely remove the lesion and simultaneously remove the ipsilateral thyroid lobe and adjacent affected structures [2]. To improve patient prognosis, capsule rupture should be avoided during the operation, and the recurrent laryngeal nerve should be retained as much as possible; preventive lymph node dissection is not recommended. The recurrence rate for PC is high, which can reach more than 50%; however, the prognosis for patients with PC is good with a long survival period. About 50% PC patients survive for more than 10 years. Patients with PC require lifelong clinical follow-up. Herein, we present two cases of PC with different secretory functions.

## Case presentations

### Case 1

**Fig. 1:**
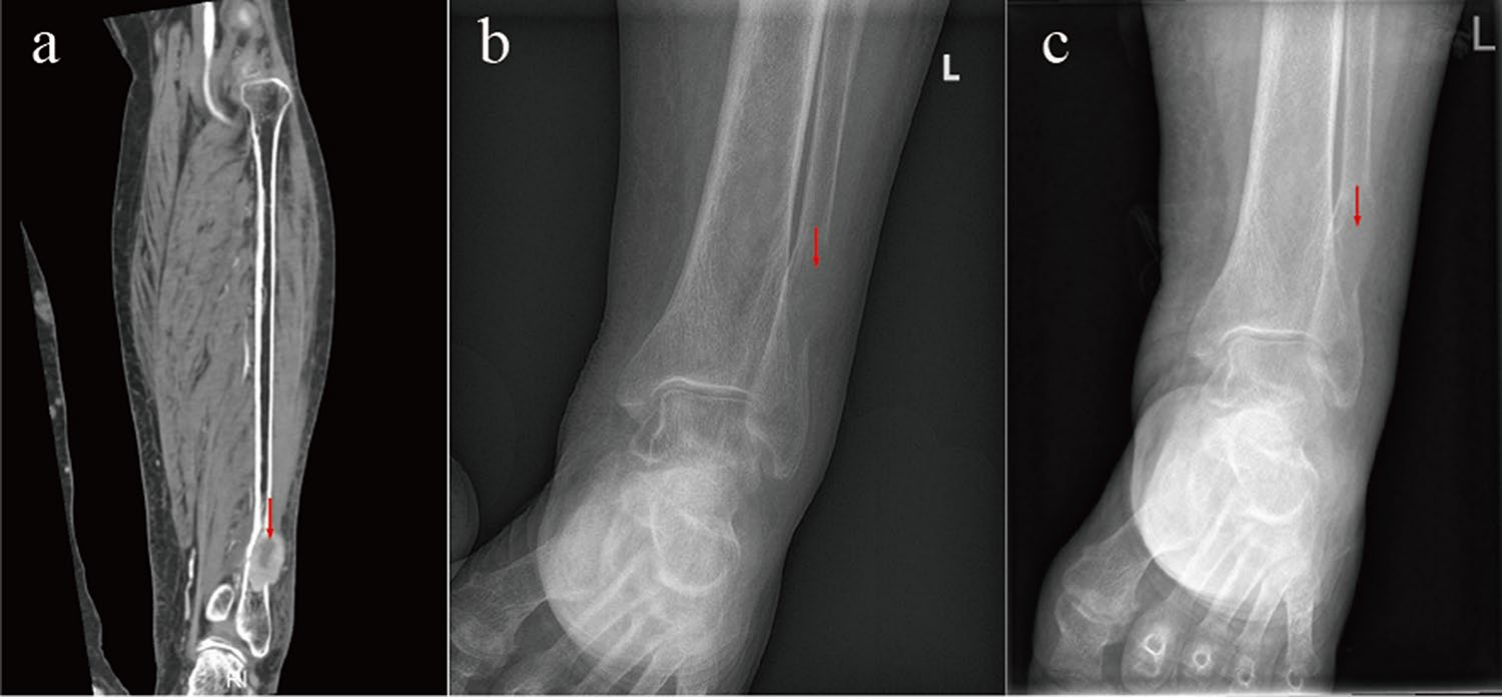
Imaging examination for Case 1. **a** MRI examination revealed a tumor in the distal left tibia. **b** X-ray examination showed a tumor in the distal left tibia. **c** X-ray examination showed that the left distal tibial tumor became smaller after operation

**Fig. 2:**
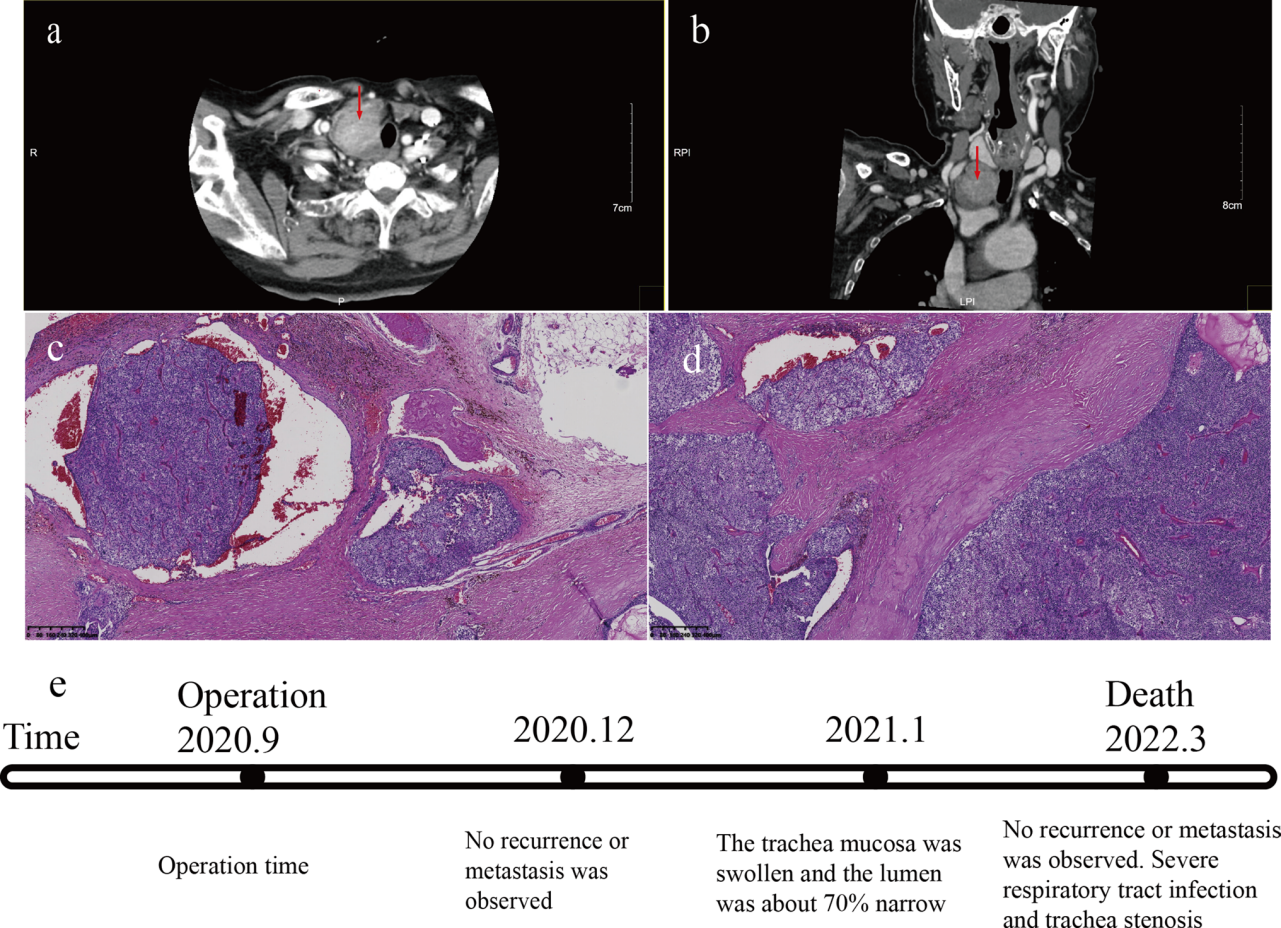
Examination and follow-up information for Case 1. **a** and **b** Computed tomography showing a mass posterior to the right inferior thyroid. **c** and **d** Hematoxylin–Eosin staining (HE), 40 × . The tumor cells are atypical; shown is the invasive growth, invading blood vessels, and penetrating the capsule. **e** Follow-up procedure

A 72-year-old woman was referred to our hospital in September 2020 with complaints of lower limb edema, pain, and walking difficulties. Lower extremity magnetic resonance imaging (MRI) revealed a tumor at distal end of left tibia, which indicated a metastatic tumor (Fig. 1a). She denied any specific disease or family history of malignancy. The biopsy results failed to identify the type and source of the tumor therefore, we recommended positron emission tomography CT (PET-CT) examination. The PET/CT showed a mass behind the right inferior lobe of the thyroid gland and an abnormal increase in glucose metabolism, which was considered as parathyroid adenoma or adenocarcinoma. Multiple instances of bone destruction were observed in the whole body with abnormal increase of glucose metabolism and the tumor at distal end of left tibia was considered as brown tumor induced by hyperparathyroidism. Ultrasonography of the neck revealed a hypoechoic mass behind the right inferior lobe of the thyroid gland, with a discernable border, uneven echo, and abundant blood flow signal. CT showed a right-posterior thyroid mass with clear boundaries, cystic changes, and push-over of adjacent thyroid tissue (Fig. 2a, b). The results of the laboratory tests were as follows: parathyroid hormone (PTH), 1425.00 pg/mL; calcium, 3.50 mmol/L; alkaline phosphatase (ALP), 1007 U/L; β2-microglobulin (β2-MG), 3.45 mg/L; and β-human chorionic gonadotropin (β-HCG), 5.9 U/L. She was preoperatively diagnosed with a parathyroid mass of an undetermined nature, which resulted in total resection of the right parathyroid tumor after surgical contraindications were eliminated. Intraoperatively, a huge mass was detected with a diameter of about 50 mm behind the right inferior lobe of the thyroid gland, which invaded the right recurrent laryngeal nerve and the right tracheal wall. Rapid pathological evaluation revealed tumor invasive growth with cellular atypia, which was consistent with PC. Therefore, total resection of the right parathyroid tumor was performed, including total resection of the right and isthmic thyroid, dissection of the central lymph nodes, and partial resection of the airway wall and the recurrent laryngeal nerve. Routine histopathology showed that the tumor had invaded the surrounding adipose tissue and blood vessels, and tumor cells penetrated the capsule; the tumor was finally diagnosed as PC (Fig. 2c, d). She recovered well after the operation; blood PTH and calcium levels returned to normal, the edema of both lower extremities improved, and the left tibial tumor was significantly reduced (Fig. 1b, c). She underwent regular follow-up visits after the operation, during which blood electrolyte and parathyroid hormone levels were checked, neck ultrasonography was reperformed, and antirespiratory infection therapy was also used. She was followed-up for 18 months after the operation, during which time no abnormalities were found on blood examination and no recurrence or metastases were found on ultrasound and CT of the neck. Nevertheless, she died of severe respiratory tract infection and tracheal stenosis in March 2022 (Fig. 2e).

### Case 2

**Fig. 3:**
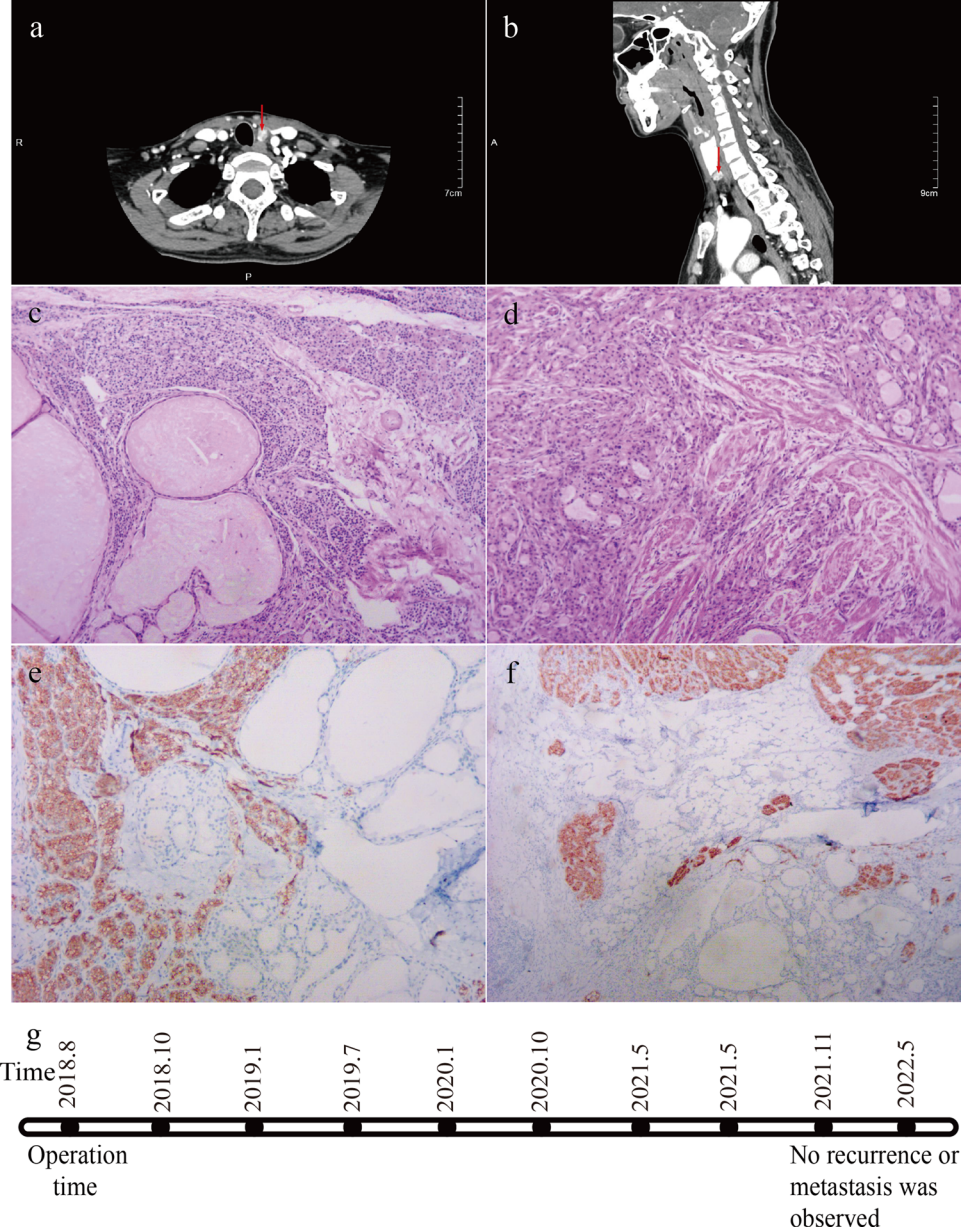
Examination and follow-up information for Case 2. **a** and **b** Computed tomography showing a nodule located below the left inferior pole of the thyroid gland. **c** and **d** Hematoxylin–Eosin staining (HE), 100 × . The tumor cells are atypical; shown is the invasive growth and invasion of the surrounding thyroid, fat, and muscle tissues. **e** and **f** (PTH, 100 ×) Immunohistochemical analysis of PTH was positive in some tumor cells. **g** Follow-up procedure

A 54-year-old woman was referred to our hospital in August 2018. Following physical examination, parathyroid nodules were detected 2 years ago. Ultrasonography of the neck showed a hypoechoic mass at the lower posterior pole of the left hypothyroid gland. CT examination of the neck revealed a dense shadow of soft tissue of the nodule below the left thyroid gland with a clear boundary (Fig. 3a, b). Blood tests did not show any abnormalities. She had a family history of parathyroid adenopathy, but denied any exposure to radiation. Surgical treatment was performed when contraindications were eliminated. During the operation, a hard mass with a diameter of about 10 mM was observed at the lower pole of the left thyroid. Complete thyroidectomy and central lymph node dissection were performed after rapid intraoperative pathology suggested that the lesion was a diffuse infiltrating thyroid follicular carcinoma. Postoperatively, she was given symptomatic treatment, and the recovery was uneventful. Routine histopathological findings suggested left PC after a consultation with the Joint Diagnostic Centre of the University of California, Los Angeles (UCLA) (Fig. 3c, d). The results of the immunohistochemical analysis were as follows: CD117, positive; GATA3, positive; CK(AE1/AE3), positive; PTH, partially positive; CgA, partially positive; SYN, weakly positive; calcitonin, negative; TG, negative; TTF1, negative; PAX8, negative; and Ki67, labeling index (LI) of < 5% (Fig. 3e, f). She was followed-up regularly after discharge, once every 3 months for the first 6 months and every 6 months thereafter. No tumor recurrence or metastases were observed as of May 2022 at the latest re-examination (Fig. 3g).

The detailed characteristics of the two patients are summarized in Table 1.

**Table 1:** Summary of characteristics of the two cases

Patients		1	2
Age		72	54
Gender		Female	Female
Complaint		Myalgia, myoedema	Nodule
Laboratory results at diagnosis	Calcium (mmol/L)	3.5	2.25
	PTH (pg/mL)	1425	56.7
	Phosphorous (mmol/L)	0.9	1.35
Last laboratory results	Calcium (mmol/L)	2.26	2.25
	PTH (pg/mL)	24.18	42
	Phosphorous (mmol/L)	1.19	1.5
Organ injury		Bone, kidney	Neg
Tumor	Localization	Right inferior	Left inferior
	Maximum diameter(mM)	50	10
Pathology	Vascular invasion	Pos	Neg
	Lymph node	Neg	Neg
	Tumor capsule invasion	Pos	Neg
	Soft tissue invasion	Pos	Pos
	CyclinD1	Limitted expression	Widely expression
Survival period	(months)	18	> 45
Follow-up results		died	alive

calcium, (2.20–2.65 mmol/L); PTH, (15-65 pg/mL); phosphorous, (0.81–1.45 mmol/L)

## Discussion

PC is a rare endocrine malignancy. The onset age of PC is usually 40–50 years, which is 10 years earlier than that of PHPT [2]. Although PHPT shows a preferentially high incidence in males, the incidence rate of PC has no correlation with sex [4]. Currently, the etiology of PC is not clear. It usually occurs sporadically and may also be observed in the context of a genetic endocrine syndrome, such as hyperparathyroidism-jaw tumor syndrome (HPT-JT), multiple endocrine neoplasia type 1 (MEN1), multiple endocrine neoplasia type 2A (MEN2A), and familial isolated hyperparathyroidism (FIHP) [5, 6]. Patient 2 had a family history of parathyroid adenopathy, which may indicate a molecular genetic basis of the disease that warrants further study. PC has been reported to be associated with radiation exposure, secondary renal failure, and grade III hyperparathyroidism [4]. The TNM staging of PC was first proposed in 1999, but a standardized TNM staging algorithm has not yet been widely accepted towing to the rarity of PC [7, 8]. The right lower parathyroid gland is a common site of PC, and the lung, liver, and bone are the most common sites of its metastases [9]. With regard to regional lymph nodes, PC mainly involves the central neck region (VI or VII region) and rarely the lateral neck (II, III, and IV regions) [10]. PC is a rare disease with inert but progressive behavior, and lymph node involvement and distant metastasis are rare at the time of initial diagnosis [6].

The clinical manifestations observed in most PC patients are usually severe symptoms and complications of PHPT, which may involve the bone, kidney, digestive tract, and nervous system. Specific symptoms are related to the target organs involved. Less than 10% of PC patients have no symptoms of PHPT. These patients have no symptoms at the early stage, but can have a neck mass at the late stage, and accompanying symptoms such as dysphagia, hoarseness, and dyspnea caused by compression or invasion of adjacent structures [5, 6]. Patient 1 presented a good postoperative prognosis for lower extremity edema, suggesting an association with secondary lesions involving the skeletal system, while patient 2 was asymptomatic preoperatively and was hence treated for abnormal physical examination, which was an incidental finding.

Laboratory examination indicated that serum PTH, calcium, and ALP of most PC patients are frequently high. PTH is usually 3–10-times higher than the upper limit of normal, calcium levels are often higher than 3.5 mmol/L, and serum alkaline phosphatase levels are always higher than 300 IU/L [1, 5]. Levels of β-HCG in the serum and urine may also be abnormally elevated [5]. Very few PC patients show no abnormalities on laboratory examination. In this report, the serum PTH, calcium, and ALP levels in patient 1 were significantly higher than normal values, while the β-HCG levels were only slightly increased. The laboratory examination of patient 2 showed no abnormalities.

Imaging examination by ultrasonography and Tc-99 m MIBI are commonly used to detect and locate PC, but they cannot distinguish between benign and malignant lesions. CT and MRI can provide information on the expansion of the lesion, the actual invasion of surrounding structures, and lymph node or persistent PC metastasis. However, the sensitivity of PC detection is low. Fluorine-18-fluorodeoxy glucose (18F-FDG)-PET-CT can evaluate an expanded area to effectively identify early metastasis and PC recurrence sites [5–7, 11, 12]. In terms of imaging, larger PC (tumor diameter: > 3 cm) present higher depth/width ratio (D/W > 1), uneven texture echoes, irregular shape, unclear boundary, thick capsule, calcification in nodules, cystic changes, and suspicious infiltration [6, 11].

**Fig. 4:**
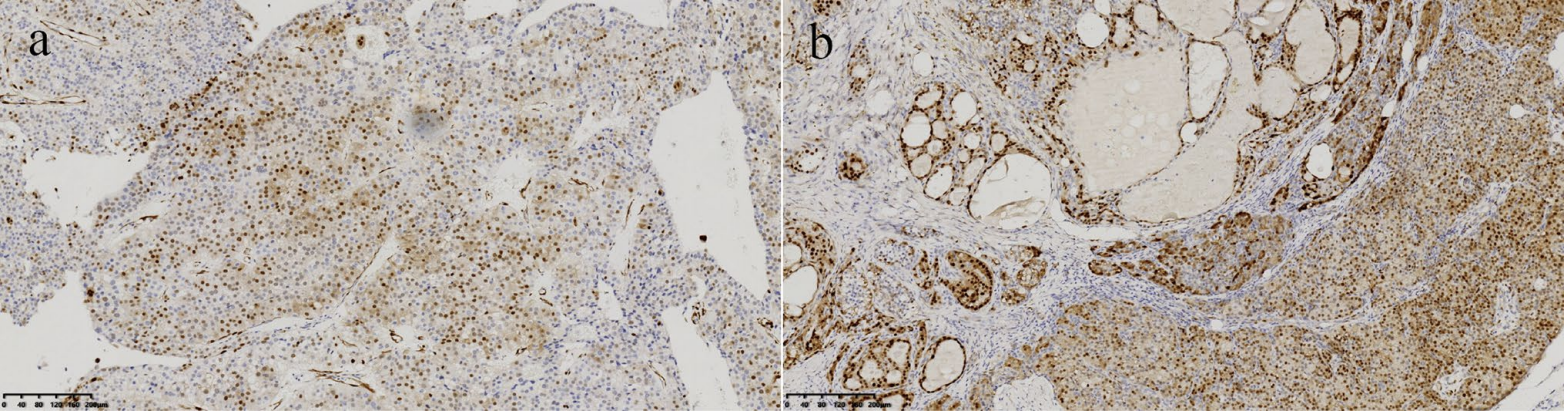
Cyclin D1 immunohistochemical staining of two PC cases. **a** Immunohistochemical analysis of cyclin D1 (100 ×) was positive in some tumor cells. **b** Immunohistochemical analysis of cyclin D1 (100 ×) was strongly positive in tumor cells

Histopathological examination of PC lacks reliable preoperative diagnostic methods. Patients with suspected PC generally do not undergo biopsy, as this may lead to capsule rupture and carries a high risk of cancer-cells spread by the needle path. The potential risk of recurrence, is also high and hence, the biopsy has limited diagnostic significance [5, 11, 13]. Because of the lack of reliable preoperative diagnosis of PC, intraoperative freezing was usually used to confirm/exclude malignant diagnosis. It had been reported that the accuracy of intraoperative frozen diagnosis of PC is low, but studies have shown that a specialized tumor center with expertise in the treatment and diagnosis of PC, combined with experienced head and neck pathologists, significantly affects the accuracy of intraoperative diagnosis [1]. Intraoperative frozen pathology is very important for surgical decision-making in PC. However, owing to the limitations of frozen sampling, it is very difficult to observe a clear basis for infiltration, and it is generally rare to report parathyroid carcinoma directly during surgery. In the absence of a clear pathological basis, surgeons may have concerns about extensive and radical resection that could lead to significant dysfunction. In previous studies, the general observation and frozen pathology report during the operation have suggested that the tissue was benign, and only the diseased gland was removed. However, postoperative paraffin pathology was suggestive of PC, which often requires a second supplementary surgery [11]. In this report, the intraoperative reports of both patients were correctly judged, based on accurate preoperative examination and communication under the Multi-Disciplinary Treatment (MDT) mode, thereby avoiding the situation of secondary surgery. The pathologist responsible for the frozen pathology integrated this information to expand the scope and capacity of intraoperative sampling, rather than only a single point of typical site sampling, which helped us to make an accurate diagnosis. Therefore, for such lesions, we believe that the communication of perfect clinical information and multidisciplinary consultation mode plays an important role in accurate intraoperative diagnosis. Accurate judgment of the nature of the mass during the operation is helpful for the choice of surgical methods, which deserves more discussion and research. Routine postoperative histopathological diagnosis is the diagnostic method of choice for PC. According to the latest WHO guidelines, the diagnosis of PC must have a clear basis for invasive growth, defined by the infiltration of surrounding structures or infiltration around blood vessels and nerves, with or without regional lymph node involvement and distant metastases [1, 9]. On visual inspection, the tumor is usually greyish white and lobulated, with a hard texture, calcification, and evidence of cystic changes on the cut surface, and adhesion to surrounding tissues. The tumor proliferates with the tumor cells forming diffuse sheets or dense nests. Microscopically, tumor cells show infiltrative growth and often invade blood vessels and surrounding tissues. The tumor cell nucleus divides actively and pathological mitosis can be seen; moreover, the nucleolus is prominent, and wide collagen band gaps and necrosis can be observed [10, 14]. If the tumor morphology conforms to PC but there is no clear evidence of invasive growth, it can be classified as atypical parathyroid adenoma (APA). Immunohistochemical analysis is helpful in the diagnosis of PC. PTH, GCM2, SYN, CgA, GATA3, and CAM5.2 are always positive in cancer cells, while TTF1, PAX8, CAL, and TG are always negative [1, 3]. Patient 2 was finally diagnosed with PC occurring in the thyroid gland. As a PC that occurs at an atypical site, simple morphology is extremely difficult to distinguish, but immunohistochemistry can help us to make a more accurate judgment of the source of the tumor. Indeed, the invasive basis remains the main evidence for the diagnosis of PC, and immunohistochemistry only plays a supporting role. For such lesions, all lesions near the capsule are considered to ensure an adequate observation field, which is the basis of correct diagnosis. Of course, this is also the principle of our actual operation. It is reported that about 90% of PC express cyclinD1 [15]. In this report, cyclinD1 staining was performed for both patients; partial expression was detected in patient 1 and overexpression in patient 2, which is consistent with the literature (Fig. 4). Fibromin, a tumor suppressor protein encoded by the CDC73 gene, is the most commonly used marker in the diagnosis of PC, with up to 75% cases of PC presenting with loss-of-function mutations of CDC73 and loss of parafibromin expression [16]. Molecular genetics has revealed that the CDC73 mutation is a main driving mutation underlying PC etiology [17]. However, the specificity of parafibromin is not sufficient as a single marker, and it should be used together with other markers to achieve a clear diagnosis of PC.

The differential diagnosis of PC includes the following: (1) Parathyroidal adenoma: a tumor composed of chief cells and transitional eosinophils, either alone or in combination. Tumor cells grow as solid sheets or in clusters. The main nucleus is densely stained, and the eosinophilic cytoplasm is vacuolated. Occasional heteromorphic nuclei are visible, and mitosis is usually low. This is a benign tumor with an intact capsule, and residual parathyroid tissue can be seen at the edge of the capsule, but there is no capsular or vascular invasion, which is also the main differentiating point. (2) Atypical parathyroid adenoma: a tumor with uncertain malignant potential. Tumor cells are more heteromorphic and mitotic than in a parathyroid adenoma, but there is no envelope or vascular invasion. (3) Secondary parathyroid tumors: tumors that spread directly from adjacent structures or metastasize and spread distantly to the parathyroid gland and generally retain the morphological and immune characteristics of the primary tumor. These can be identified by combining morphological immunohistochemical evaluation and a history of the disease. (4) Thyroid follicular tumor: thyroid follicles with glial content are usually observed on tumor morphology evaluation and the nature determination standard is consistent with that of PC, although the immunohistochemical analysis of TTF-1 is usually positive.

Surgery is the main therapeutic approach for PC, and internal medicine is used to assist in lowering blood calcium and treating complications. Studies have shown that complete resection of the lesion and the ipsilateral thyroid lobe is the gold standard for PC surgery, and the use of such methods in the initial surgery can reduce the recurrence rate. [4, 18]. Owing to the lack of appropriate preoperative diagnostic tools, imaging data and intraoperative observation are extremely important, and envelope rupture and tumor cell overflow in the surgical field should be avoided while the whole resection is performed [6]. Any uninvolved recurrent laryngeal nerve of patients should be preserved by careful dissection, while preventive dissection of lymph nodes is not recommended when there is no clear evidence of lymph node metastasis, because the literature suggests that this is not helpful for prognosis, and will rather increase the incidence rate [2, 19]. After successful surgery, patients usually develop severe hypocalcemia and hypophosphatemia, also known as “hungry bone syndrome,” which requires adequate calcium and activated vitamin D supplementation. The most common and dangerous complication of surgical treatment is recurrent laryngeal nerve (RLN) paralysis. RLN controls vocal cord movement. Therefore, flexible fiber optic laryngoscopy (FFL) is usually used to evaluate vocal cord function before and after surgery. Recent studies have found that transcutaneous laryngeal ultrasound (TLUS), as a non-invasive and painless examination, can effectively replace FFL for vocal cord assessment in specific populations [20]. Medical treatment can be divided into two parts. First, reduce and stabilize blood calcium before surgery, while treating complications; second, provide postoperative calcium and vitamin D supplementation to cope with hungry bone syndrome. There are two principles for the treatment of hypercalcemia in clinical practice, namely using normal saline expansion and loop diuretics and second, using drugs such as calcitonin, diphosphate, cinacalcet, and denosumab to inhibit bone resorption and PTH secretion [2, 6, 21]. The most common cause of death in patients with PC is hypercalcemia. Symptomatic treatment is the main clinical approach when patients with PC present with extensive metastases and surgery is not feasible. Since PC is resistant to radiotherapy, postoperative adjuvant radiation therapy has not been widely used. However, some studies have also suggested that adjuvant radiotherapy for the neck may help reduce the risk of local recurrence in patients with minimal residual parathyroid carcinoma [6, 18]. Chemotherapy has not shown efficacy in the treatment of PC [2], although radiofrequency ablation has been used to treat lung or liver metastases from functional PC [6]. In this report, patient 1 was mainly treated with surgical resection of the tumor and auxiliary calcium-lowering treatment. The tumor infiltration range of the patient was large, and part of the recurrent laryngeal nerve and trachea were removed. The late complications were obvious and the prognosis was poor. Patient 2 underwent surgical resection of the tumor and ipsilateral thyroid lobe and had good prognosis.

The recurrence rate of PC is very high. More than 50% PC patients will usually relapse within 2–3 years after the first surgical intervention, and the recurrence rate within 2–3 years is 33–78% [18, 22]. However, a long recurrence-free interval of 23 years has also been reported [18]. Positive prognostic factors related to a low recurrence rate of PC include biochemical remission, low T stage, no lymph node invasion, and a Ki-67-labeling index (LI) of < 10%, while factors with a higher recurrence rate include vascular invasion [7]. Some studies also found that the age of diagnosis and tumor size of PC were the main prognostic factors affecting survival [18], and the poor prognosis of patient 1 might be related to it. The progression of PC is slow and the long-term survival rate of patients is considerable, with the 5- and 10-year survival rates being 70–91% and 49–77%, respectively [5, 7, 15, 22]. Patients with PC should be monitored for life to identify any evidence of local recurrence or metastasis at an early stage. Blood calcium and PTH levels should be monitored every 3 months during the first 3 years, then every 6 months until the 5th year, and annually thereafter. In addition, PC patients should undergo at least one neck ultrasound examination every year [18].

In our report, one case each of functioning and nonfunctioning PC were presented. The patients' clinical symptoms, laboratory results, treatment methods, and follow-up were consistent with the literature reported. Patient 1's tumor was huge which made the total resection range large; moreover, she was old, resulting in obvious postoperative complications and poor prognosis. She eventually died of severe respiratory tract infection and tracheal stenosis. Patient 2 had the rarer non-functional type of PC. She had a long recurrence-free interval owing to prompt imaging diagnosis and complete resection of the nodule at an early stage. Taken together, we believe that the surgical scope for patients with PC is the main factor affecting the survival prognosis. Tumor size and patient age may also be related to the overall prognosis. Detection and diagnosis at an early stage, as well as complete resection of the mass are the most critical factors for determining the prognosis. Furthermore, laboratory and imaging examination can detect the mass early and hence be used for follow-up examinations.

## Data Availability

All data generated or analysed during this study are included in this published article.

## Abbreviations

CD117: C-Kit tyrosine kinase receptor
GATA3: GATA-binding protein 3
CK (AE1/AE3): Pan-cytokeratin (AE1/AE3)
TTF1: Thyroid transcription factor1
Ki-67: Kuman Ki-67 protein
CAM5.2: Anticytokeratin CAM5.2
CDC73: Cell division cycle 73
WHO: World Health Organization
SYN: Synapsin
TG: Thyroglobulin
CgA: Chromogranina
PAX8: Paired-box gene 8
GCM2: Glial cells missing 2
CAL: Calcitonin
mM: Millimeter
TNM: Tumor node metastasis
